# Prevalence, Antibiotic Susceptibility, and Molecular Characterization of *Cronobacter* spp. Isolated From Edible Mushrooms in China

**DOI:** 10.3389/fmicb.2019.00283

**Published:** 2019-02-26

**Authors:** Chengsi Li, Haiyan Zeng, Jumei Zhang, Wenjing He, Na Ling, Moutong Chen, Shi Wu, Tao Lei, Haoming Wu, Yingwang Ye, Yu Ding, Juan Wang, Xianhu Wei, Youxiong Zhang, Qingping Wu

**Affiliations:** ^1^State Key Laboratory of Applied Microbiology South China, Guangdong Provincial Key Laboratory of Microbiology Culture Collection and Application, Guangdong Open Laboratory of Applied Microbiology, Guangdong Institute of Microbiology, Guangzhou, China; ^2^Department of Food Science and Technology, Jinan University, Guangzhou, China; ^3^College of Food Science, South China Agricultural University, Guangzhou, China

**Keywords:** *Cronobacter*, prevalence, edible mushrooms, multilocus sequence typing, O-antigen serotyping, antibiotic susceptibility test

## Abstract

*Cronobacter* spp. are foodborne pathogens that can infect and cause life-threatening diseases in all age groups, particularly in infants and immunocompromised elderly. This study aimed to investigate the prevalence, antibiotic susceptibility, and molecular characteristics of *Cronobacter* spp. isolates in edible mushrooms collected from 44 cities in China. In total, 668 edible mushrooms were collected from traditional retail markets and supermarkets and were analyzed by quantitative methods, PCR-based serotyping, multilocus sequence typing (MLST), and antibiotic susceptibility testing. Among the 668 samples tested, 89 (13.32%) were positive for *Cronobacter* spp., and the contamination levels exceeded the 110 most probable number (MPN)/g in 13.48% (12/89) of the samples. *Flammulina velutipes* samples had the highest contamination rate of 17.54% (37/211), whereas *Hypsizygus marmoreus* samples had the lowest contamination rate of 3.28% (2/61). Ten serotypes were identified among 115 isolates, of which the *C. sakazakii* serogroup O1 (*n* = 32) was the primary serotype. MLST indicated that there was quite high genetic diversity in *Cronobacter* spp. and 72 sequence types were identified, 17 of which were new. Notably, *C. sakazakii* ST148 (*n* = 10) was the most prevalent, followed by *C. malonaticus* ST7 (*n* = 5). Antibiotic susceptibility testing revealed that the majority of *Cronobacter* spp. strains were susceptible to the 16 antibiotics tested. However, a portion of isolates exhibited relatively high resistance to cephalothin, with resistance and intermediate rates of 93.91 and 6.09%, respectively. One isolate (cro300A) was multidrug-resistant, with resistance to five antibiotics. Overall, this large-scale study revealed the relatively high prevalence and high genetic diversity of *Cronobacter* spp. on edible mushrooms in China, indicating a potential public health concern. To our knowledge, this is the first large-scale and systematic study on the prevalence of *Cronobacter* spp. on edible mushrooms in China, and the findings can provide valuable information that can guide the establishment of effective measures for the control and precaution of *Cronobacter* spp on edible mushrooms during production processes.

## Introduction

*Cronobacter* spp. is a gram-negative, motile, rod-shaped, non-sporulating pathogenic bacterium belonging to the family *Enterobacteriaceae* (Barron and Forsythe, 2007). Currently, the genus comprises seven species: *C. sakazakii, C. malonaticus, C. turicensis, C. universalis, C. muytjensii, C. dublinensis*, and *C. condimenti* (Iversen and Forsythe, 2007; Joseph and Forsythe, 2012; Forsythe et al., 2014). Of these, *C. sakazakii, C. malonaticus*, and *C. turicensis* have been proven relevant to human infections (Liu et al., 2013; Forsythe et al., 2014), and outbreaks have been reported in some countries (Caubilla-Barron et al., 2007; Patrick et al., 2014). *Cronobacter* infections occur in all age groups, although with a greater incidence in the very young and elderly, who are typically more immunocompromised (Forsythe S., 2018). Clinical symptoms of infection in infants include meningitis, bacteraemia, and necrotizing enterocolitis, with mortality rates of 40–80% (Friedemann, 2009). Infections in the adults show a wide range of symptoms: conjunctivitis, biliary sepsis, urosepsis, wound infections, appendicitis, and pneumonia (Lai, 2001; Patrick et al., 2014). *Cronobacter* spp. has been isolated not only from clinical samples, but also from various foods, including cereals, meat, herbs, spices, salads, fruits, and vegetables, as well as their as well as derivative food products (Alsonosi et al., 2015; Ueda, 2017). *Cronobacter* spp. infecting infants are generally believed to be derived from infant milk formulas (van Acker et al., 2001), although other food sources, such as breast milk, have been suspected in some cases (Stoll et al., 2004). The source of infections in adults is still unidentified (Joseph and Forsythe, 2012). Some research reports indicated that the principal sources of this organism are associated with soil, water, and vegetables (Ueda, 2017; Zeng et al., 2018b). However, a clear understanding about the epidemiology and reservoirs of *Cronobacter* spp. is still lacking (Holý and Forsythe, 2014). An in-depth understanding of the genetic diversity of *Cronobacter* spp. can effectively promote reliable source tracking of contaminated foods and enhance the resolution of surveillance.

PCR-based O-antigen serotyping methods for identifying isolates in epidemiological studies of bacteria have been successfully developed (Jones et al., 2012). To date, 24 serogroups of *Cronobacter* spp. have been identified (BlaŽková et al., 2015; Forsythe S. J., 2018). Furthermore, multilocus sequence typing (MLST) based on seven housekeeping genes (*atpD, fusA, glnS, gltB, gyrB, infB*, and *ppsA*) has been established in the genus *Cronobacter*. The *Cronobacter* PubMLST database (http://pubmlst.org/Cronobacter/) is an open access, sequence-based repository in which 633 sequence types (STs) are stored. MLST has been proven a powerful tool to effectively identify and discriminate different *Cronobacter* species, and its application has accelerated our understanding of the STs and studies on outbreaks of these bacteria (Joseph et al., 2012b; Xu et al., 2015). *C. sakazakii* ST4 is the predominant ST in the clinical setting (Joseph and Forsythe, 2011; Hariri et al., 2013). *C. malonaticus* ST7 is significantly associated with adult infections, although the source has not yet been determined (Joseph and Forsythe, 2012). Till now, some novel STs were also reported to cause infant meningitis (Cui et al., 2017; Chaves et al., 2018; Zeng et al., 2018a).

Currently, antimicrobial resistance, particularly multidrug resistance, poses a public health concern because certain pathogenic bacteria are not disrupted by conventional treatment, which results in prolonged illness and a greater risk of death. Most *Cronobacter* spp. isolates are susceptible to commonly used antibiotic agents; however, prolonged and extensive antibiotic use has led to the emergence of single- and multidrug resistant strains. Of late, some *Cronobacter* spp. isolates from food have been reported to be resistant to cephalothin, cefotaxime, streptomycin, ampicillin, penicillin G, and amoxicillin-clavulanate (Chon et al., 2012; Pan et al., 2014; Fei et al., 2017a). Therefore, it is necessary analyze the potential association between antibiotic resistance and the source of *Cronobacter* spp. isolates, especially the clinical setting and various foods.

In China, edible mushrooms are popular foods that are frequently used as raw materials in other foods owing to their high nutritional value, delicacy, and good chewiness (Chang and Buswell, 1996; Bao et al., 2013; Ye et al., 2014). Although infants are not fed with mushroom, the elderly (or adults) will eat it, despite the potential risk (Chon et al., 2012; Aksu et al., 2016; Chitrakar et al., 2018). A recent study reported an acute gastroenteritis outbreak by the infection of *C. sakazakii* which occurred in a local senior high school of China, although without any specification about the food (Yong et al., 2018). In addition, China's share in global mushroom production increased from 5.7% in 1978 to 80% in 2011 (Zhang et al., 2014). However, our previous study showed that edible mushrooms are often contaminated with foodborne pathogens, such as *Listeria monocytogenes, Staphylococcus aureus*, and *Cronobacter* (Chen et al., 2014; Ye et al., 2014; Wu et al., 2015; Huang et al., 2018). These findings highlight the importance of evaluating the microbiological profile of edible mushrooms for food safety. Therefore, this study aimed to investigate the contamination levels of edible mushrooms in China with *Cronobacter* spp., to identify molecular features of the isolates by O-antigen serotyping and MLST analysis, and to evaluate antibiotic resistance patterns.

## Materials and Methods

### Sampling

**Figure 1:**
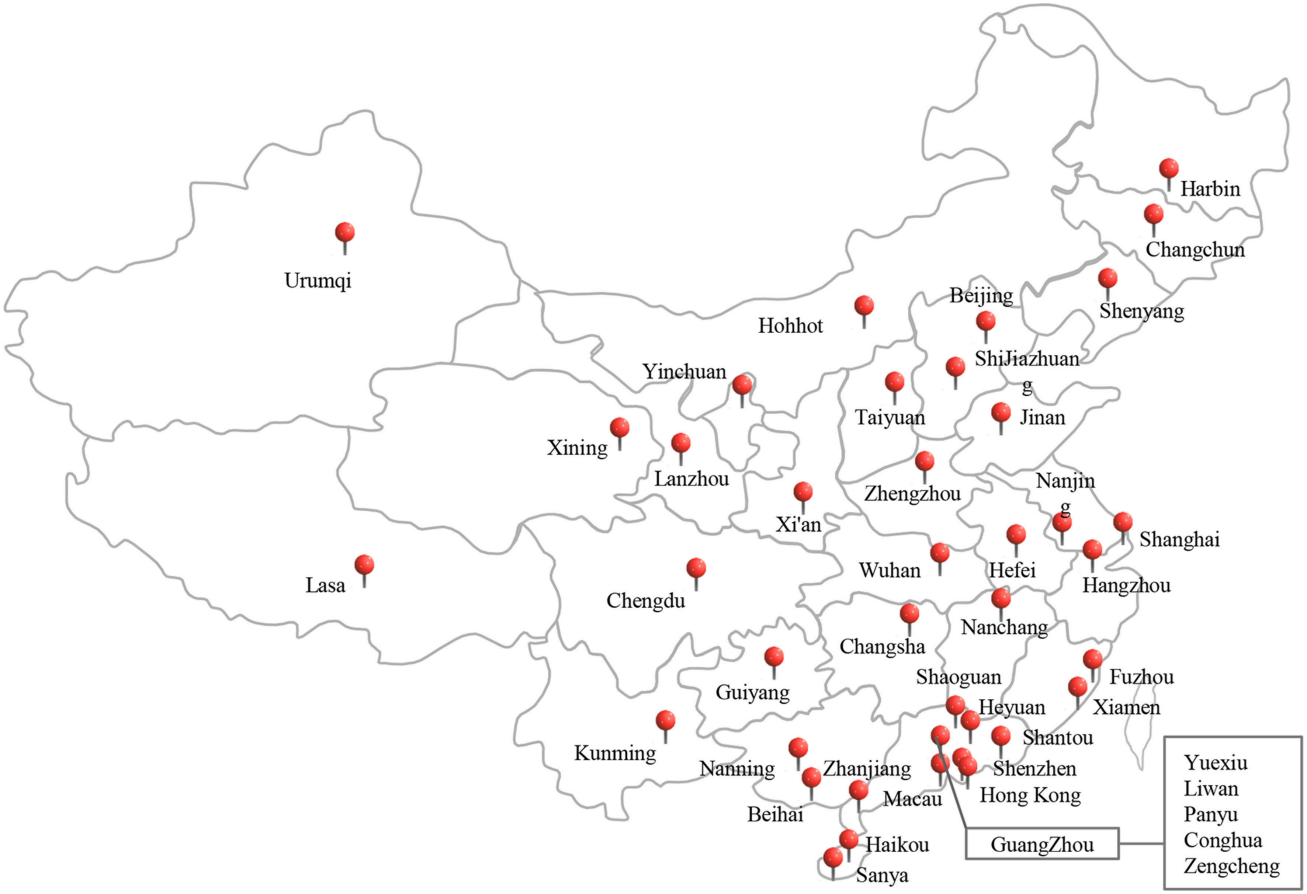
The sampling locations of the edible mushrooms for this study in China, including 44 cities, covering most cities of China.

In total, 668 edible mushroom samples, including *Flammulina velutipes* (*n* = 211), *Lentinus edodes* (*n* = 114), *Pleurotus ostreatus* (*n* = 104), *Pleurotus eryngii* (*n* = 98); *Hypsizygus marmoreus* (*n* = 80), and other species (*n* = 61) were collected from traditional retail markets and supermarkets during July 2011 and June 2016 (Table 1). The samples were obtained from 44 cities geographically spread over China (Figure 1). The samples were placed in sterile sealed plastic bags, transferred under cold conditions (below 4°C) to the laboratory, and analyzed immediately.

**Table 1:** Prevalence and contamination levels of *Cronobacter* spp. on edible mushrooms.

**Sample**	**Prevalence rate (%)**	**MPN value (MPN/g)**			**Positive sample contamination level (MPN/g)**	**Total sample contamination level (MPN/g)**
		**<10 (%)**	**10 to 110 (%)**	**110 ≤ (%)**		
*Flammulina velutiper*	37/211 (17.54)	25/37 (67.57)	7/37 (18.92)	5/37 (13.51)	21.43	3.76
*Lentinus edodes*	6/114 (5.26)	6/6 (100.00)	0/6 (0)	0/6 (0)	1.18	0.06
*Pleurotus ostreatus*	15/104 (14.42)	15/15 (100.00)	0/15 (0)	0/15 (0)	1.88	0.27
*Pleurotus eryngii*	10/98 (10.20)	10/10 (100.00)	0/10 (0)	0/10 (0)	0.23	0.02
*Hypsizygus marmoreus*	4/80 (5.00)	2/4 (50.00)	0/4 (0)	2/4 (50.00)	55.81	2.79
*Others	17/61 (27.87)	11/17 (64.71)	1/17 (5.88)	5/17 (29.41)	34.28	9.55
Total	89/668 (13.32)	69/89 (77.53)	8/89 (8.99)	12/89 (13.48)	18.60	2.45

### Isolation and Identification of *Cronobacter* spp.

Quantitative detection was conducted by an isolation and enrichment method according to the National Food Safety Standard of China Food microbiological examination: *Enterobacter sakazakii* (GB 4789.40-2010; National Standard of the People's Republic of China, 2010) for powdered infant formula (PIF), as described previously, with some modifications (Xu et al., 2015; Ling et al., 2018). We modified the method based on the analysis of PIF to mushroom for *Cronobacter*. The nine-tube method was applied to calculate the most probable number (MPN). The MPN was determined on the basis of the number of positive tube(s) in each of the three sets and the MPN table (GB 4789.7-2013, National Standard of the People's Republic of China, 2013). Green or blue-green colonies in chromogenic *Enterbacter sakazakii* Agar Plate were considered as presumptive *Cronobacter* spp. and were selected for analysis using API 20E diagnostic strips (BioMérieux, Marcy-l'Étoile, France). Species identification of these isolates was conducted by *fusA* sequencing (Joseph and Forsythe, 2012).

### PCR-Based O-Antigen Serotyping

Genomic DNA was extracted with the HiPure Bacterial DNA Kit (Magen Technologies, Guangzhou, China). The serotypes of *Cronobacter* spp. isolates were identified according to previously reported *Cronobacter* molecular serotyping schemes, including *C. sakazakii* O1 to O4, O6, and O7 (Jarvis et al., 2011; Sun et al., 2011, 2012), *C. malonaticus* O1 to O4 (BlaŽková et al., 2015), and *C. dublinensis* O1 to O4 (Jarvis et al., 2011).

### Multilocus Sequence Typing and Sequence Analysis

MLST was used for molecular typing of the *Cronobacter* isolates, as previously reported (Joseph et al., 2012a). The designation of new alleles and STs was verified by Stephen J. Forsythe (https://pubmlst.org/cronobacter/), the MLST database curator. Neighbor-joining trees were constructed based on the seven MLST loci (concatenated length of 3,036 bp) of the *Cronobacter* spp. using MEGA (version 5.05) as previous study (Ling et al., 2018).

### Antimicrobial Susceptibility Testing

The susceptibility profiles of *Cronobacter* spp. isolates were determined by antimicrobial dilution and disk susceptibility testing using Mueller-Hinton agar (Huankai, Guangzhou, China), following the protocols of the Clinical and Laboratory Standards Institute (Jorgensen, 2015). Sixteen antimicrobials (AMs) (Oxoid, Hampshire, United Kingdom) recommended for *Enterobacteriaceae* were tested, as previously reported (Ling et al., 2018).

## Results

### Isolation and Contamination Levels of *Cronobacter* spp. on Edible Mushrooms

As shown in Table 1, the 668 samples were isolated from six mushroom species, and 89 (13.32%) samples tested positive for *Cronobacter* spp. The prevalence rate of *Cronobacter* spp. varied among the different mushroom species: 17.54% (37/211) were obtained from *F. velutipes*, 5.26% (6/114) from *L. edodes*, 14.42% (15/104) from *P. ostreatu*s, 10.20% (10/98) from *P. eryngii*, 5.00% (4/80) from *H. marmoreus*, and 27.87% (17/61) from other species. Based on the MPN analysis, the contamination level of *Cronobacter* spp. was <10 MPN/g in 77.53% (69/89) of the samples, ranged between 10 and 110 MPN/g in eight samples, and exceeded 110 MPN/g in 12 samples, five of which were *F. velutipes* samples. Moreover, the positive samples, including *H. marmoreus* (55.46 MPN/g), *V. volvacea* (50.29 MPN/g), and *F. velutipes* (21.43 MPN/g) showed a higher mean contamination level than other species of mushroom. The mean contamination level of *L. edodes, P. ostreatus*, and *P. eryngii* was lower than 2 MPN/g. Isolates from the same sample belonging to the same ST and serotype were considered clonal. Thus, the 115 *Cronobacter* isolates from the 89 positive samples were identified as three species (Table 2). The majority (60.87%, 70/115) of the isolates were identified as *C. sakazakii*, followed by *C. malonaticus* (29.57%, 34/115), and *C. dublinensis* (9.57%, 11/115).

**Table 2:** Species and serotypes of *Cronobacter* spp. isolates in this study.

**Species**	**No. of isolates**	**Serotype**	**No. of isolates**
*C. sakazakii*	70	O1	32
		O2	20
		O3	11
		O4	4
		O7	3
*C. malonaticus*	34	O1	10
		O2	15
		O3	9
*C. dublinensis*	11	O1	9
		O2	2

### Serotyping

O-antigen serotyping according to the size of the target gene was employed to evaluate the distribution of O-antigen serotypes among the 115 *Cronobacter* spp. isolates from edible mushrooms. As shown in Table 2, all *C. sakazakii* serotypes were detected among the 70 isolates. Among them, O1 was the dominant serotype (32 isolates), followed by serotype O2 (20 isolates). In addition, 34 *C. malonaticus* isolates were classified into serotypes O1 (10 isolates), O2 (15 isolates), and O3 (9 isolates). Nine *C. dublinensis* serotype O1 and 2 serotype O2 isolates were identified.

### MLST Sequence Analysis

**Figure 2:**
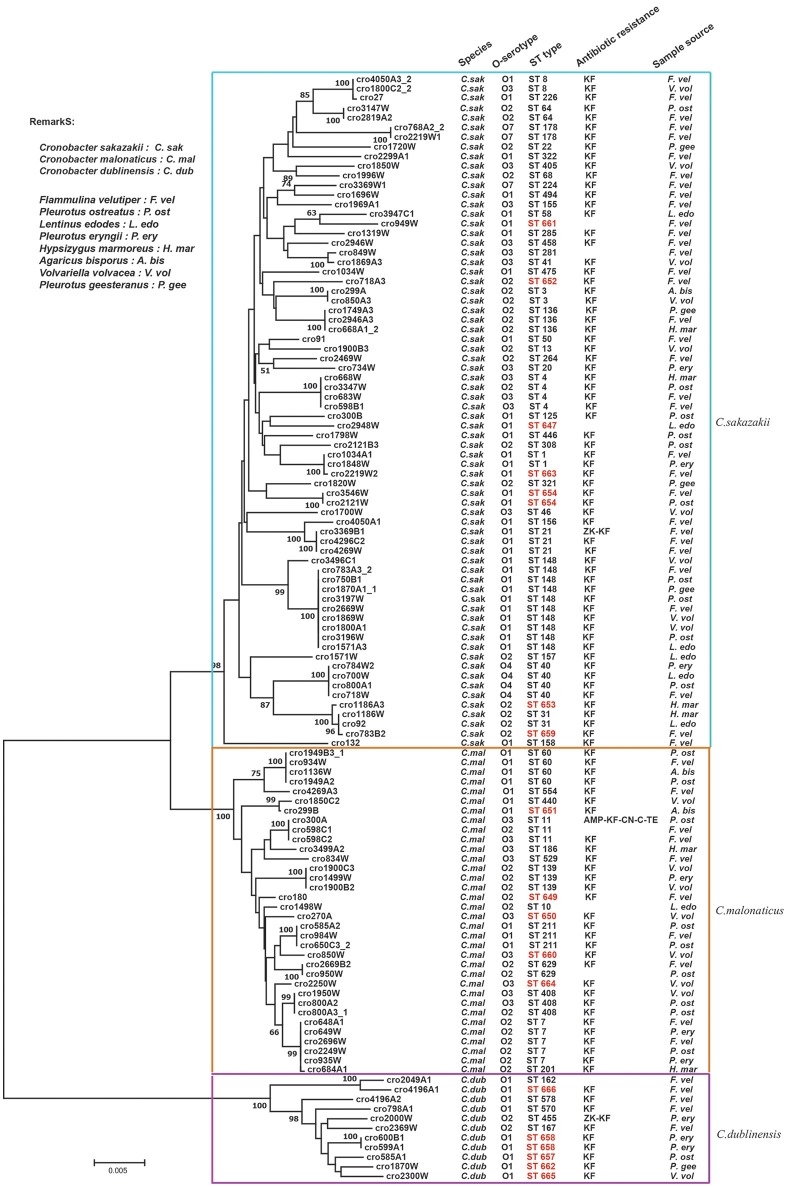
Neighbor-joining phylogenetic tree based on the seven MLST loci (3,036 base pair concatenated length) of *Cronobacter* spp. isolates. This tree was generated using MEGA (version 5.05) with 1,000 bootstrap replicates.

A phylogenetic tree was constructed by the neighbor-joining method using the concatenated sequences of seven housekeeping genes (3,036 bp) for the 115 *Cronobacter* isolates. As shown in Figure 2, the tree showed a clear relatedness between the 115 *Cronobacter* spp. isolates, which were grouped into 72 STs, 17 of which were novel, and divided into three clusters representing the three species The new nucleotide sequences have been deposited in the PubMLST database under IDs 2457 to 2474 (http://pubmlst.org/Cronobacter/). Fifty-two of the 72 STs were unique to only one isolate, and the remaining 20 STs covered two to 10 isolates each. There were 44 STs including seven new STs in *C. sakazakii* isolates. ST148 was the dominant ST (*n* = 10), followed by ST4 (*n* = 4), and ST40 (*n* = 4). Eighteen STs were included in *C. malonaticus* isolates and ST7 (*n* = 5) was the dominant ST, followed by ST60 (*n* = 4). In *C. dublinensis* isolates, the newly identified ST658 (*n* = 2) was the dominant ST, the other isolates had unique STs. Furthermore, ST1 was found only in *F. velutipes* and *P. eryngii*; ST4 was found in *F. velutipes, P. ostreatus*, and *H. marmoreus*; ST7 was found in *F. velutipes, P. eryngii*, and *V. volvacea*; ST8 was found in *F. velutipes* and *V. volvacea*; ST60 was found in *F. velutipes, P. ostreatus*, and *Agaricus bisporus*, and ST64 was found in *F. velutipes* and *P. ostreatus*. Several pathogenic STs were found in *F. velutipes*.

### Antimicrobial Susceptibility Testing

The 115 *Cronobacter* spp. isolates were subjected to 16 antimicrobial susceptibility tests (Table 3). All isolates were susceptible to TOB, SAM, FEP, CRO, CIP, IPM, SXT, ATM, and AMC, and there was no significant difference among *C. sakazakii, C. malonaticus, and C. dublinensis* isolates. The majority of isolates were susceptible to AMP, KZ, CN, AK, C, and TE, with sensitivity rates of 99.13, 72.17, 99.13, 97.39, 99.13, and 99.13%, respectively. The tests revealed that 93.91 and 6.09% of isolates exhibited resistance and intermediate to KF, respectively. One *C. malonaticus* isolate (cro300A) was detected as multidrug-resistant strains with resistance to five AMs (AMP-KF-CN-C-TE).

**Table 3:** Antimicrobial resistance profiles of 115 *Cronobacter spp*. Isolates.

**Antimicrobial group**	**Antibiotic**	**Disk code**	**Antimicrobial class^**a**^ according to the WHO**	**No. (%) of** ***C.sakazakii*** **(*****n*** **=** **70)**			**No. (%) of** ***C.malonaticus*** **(*****n*** **=34)**			**No. (%) of** ***C.dublinensis*** **(*****n*** **=** **11)**			**No. (%) of** ***Cronobacter spp***. **(*****n*** **=** **115)**		
				**R**	**I**	**S**	**R**	**I**	**S**	**R**	**I**	**S**	**R**	**I**	**S**
Penicillins	Ampicillin	AMP	CI	0 (0)	0 (0)	70 (100.00)	1 (2.94)	0 (0)	33 (97.06)	0 (0)	0 (0)	11 (100.00)	1 (0.87)	0 (0)	114 (99.13)
	Ampicillin/sulbactam	SAM	CI	0 (0)	0 (0)	70 (100.00)	0 (0)	0 (0)	34 (100.00)	0 (0)	0 (0)	11 (100.00)	0 (0)	0 (0)	115 (100.00)
	Amoxicillin/clavulanic	AMC	CI	0 (0)	0 (0)	70 (100.00)	0 (0)	0 (0)	34 (100.00)	0 (0)	0 (0)	11 (100.00)	0( 0)	0 (0)	115 (100.00)
Cephalosporins	Cefepime	FEP	CI	0(0)	0 (0)	70 (100.00)	0 (0)	0 (0)	34 (100.00)	0 (0)	0 (0)	11 (100.00)	0 (0)	0 (0)	115 (100.00)
	Ceftriaxone	CRO	CI	0 (0)	0 (0)	70 (100.00)	0 (0)	0 (0)	34 (100.00)	0 (0)	0 (0)	11 (100.00)	0 (0)	0 (0)	115 (100.00)
	Cefazolin	KZ	HI	1 (1.43)	24 (34.29)	45 (64.29)	0 (0)	1 (2.94)	33 (97.06)	1 (9.09)	5 (45.45)	5 (45.45)	2 (1.74)	30 (26.09)	83 (72.17)
	Cephalothin	KF	HI	67 (95.71)	3 (4.29)	0 (0)	31 (91.18)	3 (8.82)	0 (0)	10 (90.91)	1 (9.09)	0 (0)	108 (93.91)	7 (6.09)	0 (0)
Aminoglycosides	Gentamicin	CN	CI	0 (0)	0 (0)	70 (100.00)	1 (2.94)	0 (0)	33 (97.06)	0 (0)	0 (0)	11 (100.00)	1 (0.87)	0 (0)	114 (99.13)
	Tobramycin	TOB	CI	0 (0)	0 (0)	70 (100.00)	0 (0)	0 (0)	34 (100.00)	0 (0)	0 (0)	11 (100.00)	0 (0)	0 (0)	115 (100.00)
	Amikacin	AK	CI	0 (0)	2 (2.86)	68 (97.14)	0 (0)	1 (2.94)	33 (97.06)	0 (0)	0 (0)	11 (100.00)	0 (0)	3 (2.61)	112 (97.39)
Quinolones	Ciprofloxacin	CIP	CI	0 (0)	0 (0)	70 (100.00)	0 (0)	0 (0)	34 (100.00)	0 (0)	0 (0)	11 (100.00)	0 (0)	0(0)	115 (100.00)
Carbapenems	Imipenem	IPM	CI	0 (0)	0 (0)	70 (100.00)	0 (0)	0 (0)	34 (100.00)	0 (0)	0 (0)	11 (100.00)	0 (0)	0 (0)	115 (100.00)
Sulfonamides	Trimethoprim/sulfamethoxazole	SXT	HI	0 (0)	0 (0)	70 (100.00)	0(0)	0 (0)	34 (100.00)	0 (0)	0 (0)	11 (100.00)	0 (0)	0 (0)	115 (100.00)
Monobactams	Aztreonam	ATM	HI	0 (0)	0 (0)	70 (100.00)	0(0)	0 (0)	34 (100.00)	0 (0)	0 (0)	11 (100.00)	0 (0)	0 (0)	115 (100.00)
Amphenicols	Chloramphenicol	C	HI	0 (0)	0 (0)	70 (100.00)	1 (2.94)	0 (0)	33 (97.06)	0 (0)	0 (0)	11 (100.00)	1 (0.87)	0 (0)	114 (99.13)
Tetracyclines	Tetracycline	TE	HI	0 (0)	0 (0)	70 (100.00)	1 (2.94)	0 (0)	33 (97.06)	0 (0)	0 (0)	11 (100.00)	1 (0.87)	0 (0)	114 (99.13)

a * CI, critically important; HI, highly important; I, important; R, Resistant; I,Intermediate; S, Susceptible*.

## Discussion

*Cronobacter* spp. are well known to be opportunistic foodborne pathogens that can cause infections and life-threatening diseases in all age groups, especially in infants and the elderly, who are immunocompromised. In this long-term and large-scale study, 668 edible mushroom samples were analyzed, and the overall prevalence of *Cronobacter* spp. was determined to be 13.32% (89/668). Based on comparison with other large-scale and systematic investigations in China, the prevalence of *Cronobacter* spp. on edible mushrooms is lower than that in ready-to-eat foods (18.6%, 52/280) (Xu et al., 2015) and raw vegetables (30.27%, 122/403) (Ling et al., 2018). The prevalence of *Cronobacter* spp. in *F. velutipes* was very high 17.54% (37/211). In our previous study, we frequently detected *Listeria monocytogenes* on edible mushrooms, particularly in *F. velutipes* (Chen et al., 2014), for which the contamination rate was 55.6% (Wu et al., 2015). Therefore, it is necessary to pay close attention to this correlation between *Cronobacter* spp. and *F. velutipes*. The contamination rate in the other mushroom species was not analyzed because of the limited number of samples for each species, but it is worth noting the high contamination rate of *V. volvacea* (11/17) (detail not shown). Usually, edible mushrooms require relatively high temperature (28–35°C) and high humidity to grow; conditions that permit bacterial growth (Bao et al., 2013). *V. volvacea* were largely cultivated in soil, whereas the other mushroom species were mainly grown in plastic bags and thus had no direct contact with the soil (Chang, 1978; Zhang et al., 2014). Some studies have shown that the soil maybe a principal source of *Cronobacter* spp. (Ueda, 2017). Moreover, the base growth material for *V. volvacea* was generally not sterilized. Furthermore, *V. volvacea* usually was stored in bulk at ambient temperature, which promotes the growth of pathogens (Bao et al., 2013). These might be reasons for the high contamination rate of *Cronobacter* spp. in *V. volvacea*. Future studies should include a larger sample size of *V. volvacea* and establish a continuous surveillance of *Cronobacter* spp. for reliable determination of the contamination status. Even low levels of *Cronobacter* spp. in powdered infant formula are considered to be a risk factor, which suggests that our result also indicate a potential risk for the immunocompromised. Although edible mushrooms are not ready-to-eat foods, they can potentially cause cross contamination of the environment and other foods (Kilonzo-Nthenge et al., 2012). To date, no standard permissible limit of *Cronobacter* spp. on edible mushrooms has been established in China. The establishment of a definite microbiological standard to ensure the quality of edible mushrooms products is essential for food safety in China.

In this study, 10 *Cronobacter* spp. serogroups were identified in the 115 isolates. *C. sakazakii* serotype O1 (32/70 isolates) was the most prevalent, followed by *C. sakazakii* serotype O2 (20/70 isolates). This finding was in agreement with our previous survey in raw vegetables (Ling et al., 2018), but in disagreement with previous studies, in which *C. sakazakii* serotype O2 was the most dominant serotype in ready-to-eat foods in China (Xu et al., 2015) and powdered infant formula (PIF) (Fei et al., 2017b). It is worth noting that both serotypes O1 and O2 have been associated with clinical sources (Scharinger et al., 2017).

Approximately 30% of isolates in the *Cronobacter* PubMLST database are from China (Forsythe et al., 2014); the identification of 17 new STs in this study indicates that further studies on genetic characteristic of *Cronobacter* in China are necessary, especially those from infections with unknown sources. This would help developing control measures for this pathogen and future epidemiological studies. Notably, *C. sakazakii* ST148 was the most dominant ST. Although ST148 have not reported to associated with human infection, one *C. sakazakii* ST148 strain was isolated from blood of a 64-year-old in Denmark in 2009 according to PubMLST database (http://pubmlst.org/Cronobacter/). Some pathogenic STs (e.g., ST1, ST4, ST7, ST8, ST60, and ST64) were also identified in this study.

Antibiotic susceptibility tests revealed that the isolates from edible mushrooms were susceptible to most antibiotics. However, a portion of isolates exhibited high or intermediate resistance to cephalothin, cefazolin, cephalothin, and amikacin, which was basically consistent with previous reports (Lai, 2001; Molloy et al., 2009; Chon et al., 2012). It has been reported that some isolates that exhibit intermediate resistance can become resistant under certain circumstances (Ruiz-Bolivar et al., 2011). β-Lactamases and extended spectrum-β-lactamases (ESBL)-related genes have already been reported in *Cronobacter* spp. (Girlich et al., 2001; Caubilla-Barron et al., 2007; Müller et al., 2014). ESBL confer resistance to penicillins, third-generation cephalosporins, and monobactams (Vasconcellos et al., 2018). Most clinical *Cronobacter* spp. isolates are multidrug-resistant (Cui et al., 2017; Shi et al., 2017; Zeng et al., 2018a), whereas isolates from foods generally have low drug resistance. It is still unclear whether multi-drug resistance is acquired by *Cronobacter* before or after infections in the adaptation process. Therefore, a comprehensive study of the molecular mechanisms of antibiotic resistance and correlation analysis of isolates from various environments, foods, and clinical settings should be explored. Clearly, over- and misuse of antimicrobial agents should be avoided, and the transmission and transfer of resistance genes should be closely evaluated.

## Conclusion

In this study, it is necessary to pay more attention to the correlation between *Cronobacter* spp. and *F. velutipes* or *V. volvacea* with the high contamination rates of *Cronobacter* spp. Detection of pathogenic *Cronobacter* STs strongly implicates that this could be a potential risk to humans. Although edible mushrooms are not ready-to-eat foods, they can potentially cause cross contamination of the environment and other foods during transport, sales, and food preparation processes (Kilonzo-Nthenge et al., 2012). The correlation analysis among foodborne bacteria isolates from environments, foods, and clinics need to be established for identifying the route of transmission and surveillance of multi-drug resistance of *Cronobacter* spp.

## Author Contributions

CL, HZ, and JZ contributed to this work equally. QW and HZ conceived and designed the experiments. CL, WH, NL, MC, SW, HW, YY, XW, and YZ performed the experiments. CL, HZ, JZ, YD, and JW analyzed the data. HZ and WH drafted the manuscript. QW supervised the project. All authors read and approved the final manuscript.

### Conflict of Interest Statement

The authors declare that the research was conducted in the absence of any commercial or financial relationships that could be construed as a potential conflict of interest.
